# Chimeric antigen receptor T-cell therapy in neuroimmune diseases: mechanistic rationale, clinical experience, and translational challenges

**DOI:** 10.3389/fimmu.2026.1913956

**Published:** 2026-09-16

**Authors:** Yaqiao Yi, Hui Xiao, Yitong Yin, Rong Zeng, Qingqing Yan, Yonghe Wu, Kailin Yang, Guilong Fu, Hui Xu, Xiongmei Zhang

**Affiliations:** 1 School of Traditional Chinese Medicine, Hunan University of Chinese Medicine, Changsha, Hunan, China; 2 Institute of Basic Research in Traditional Chinese Medicine, Hunan Academy of Chinese Medicine, Changsha, Hunan, China; 3 People’s Hospital of Ningxiang City, Hunan University of Chinese Medicine, Changsha, China; 4 School of Traditional Chinese Medicine, Hunan Traditional Chinese Medical College, Zhuzhou, Hunan, China; 5 School of Artificial Intelligence, Sun Yat-sen University, Zhuhai, China

**Keywords:** autoimmune encephalitis, chimeric antigen receptor T-cell therapy, multiple sclerosis, myasthenia gravis, myelin oligodendrocyte glycoprotein antibody-associated disease, neuroimmune diseases, neuromyelitis optica spectrum disorder, Stiff-Person syndrome

## Abstract

Neuroimmune diseases comprise a heterogeneous group of disorders in which B cells, plasmablasts, plasma cells, autoantibodies, and T cells contribute differently to tissue injury. Although B-cell-directed biologics have improved disease control, some patients continue to experience treatment-refractory activity, relapse after treatment withdrawal, or cumulative neurological disability. Chimeric antigen receptor T-cell (CAR-T cell) therapy offers a means of achieving deeper and potentially sustained depletion of defined pathogenic immune-cell compartments. This narrative review integrates the mechanistic rationale, target selection, platform design, published human experience, and translational challenges of CAR-T cell therapy in neuroimmune diseases. Current strategies predominantly target CD19-expressing B-lineage cells or BCMA-expressing antibody-secreting cells; these therapeutic targets are distinct from disease autoantigens, and their relevance depends on the immunopathology of each disorder. CAR design, persistence, lymphodepletion, and the use of integrating or transient expression platforms further influence treatment activity and risk. Clinical development remains uneven across indications. Myasthenia gravis has the most diverse clinical experience, encompassing integrating CD19-, BCMA-, and dual CD19/BCMA-targeted products, together with a transient BCMA-targeted mRNA platform evaluated in a randomized, placebo-controlled phase 2b study. In neuromyelitis optica spectrum disorder, an open-label phase 1 study has demonstrated early clinical and immunological activity of BCMA-targeted CAR-T cells. Evidence in multiple sclerosis and stiff-person syndrome is derived from individual reports, small early-phase studies, and conference data, whereas experience in other neuroimmune diseases remains limited to isolated reports or small case series. Across indications, interpretation is constrained by small and heterogeneous cohorts, limited follow-up, and the predominance of uncontrolled observations. Major translational considerations include target breadth and selectivity, the contribution of lymphodepletion, the disease-dependent relevance of central nervous system trafficking, acute and delayed toxicity, patient selection, manufacturing feasibility, biomarker development, and standardized long-term outcome assessment. CAR-T cell therapy has shown preliminary clinical activity in selected patients with refractory neuroimmune diseases, but its comparative efficacy, durability, and long-term safety remain undefined. It therefore remains investigational for most neuroimmune indications.

## 1 Introduction

Neuroimmune diseases represent a heterogeneous group of disorders defined by pathological interactions between the immune system and the central nervous system (CNS), the peripheral nervous system, and the neuromuscular junction. This spectrum includes multiple sclerosis (MS), neuromyelitis optica spectrum disorder (NMOSD), myasthenia gravis (MG), anti-N-methyl-D-aspartate receptor encephalitis (NMDARE), stiff-person syndrome (SPS), myelin oligodendrocyte glycoprotein antibody-associated disease (MOGAD), and other immune-mediated neurological disorders. Although these diseases share immune-mediated mechanisms of tissue injury, they differ substantially in the relative contributions of autoantibodies, B cells, plasma cells, T cells, and tissue-resident immune responses. Therapeutic strategies have consequently evolved from non-specific immunosuppression toward disease- and pathway-directed biologics, including B cell-targeted therapies (1, 2). B cells occupy a central position in the pathogenesis of many of these diseases. They drive tissue injury by producing pathogenic autoantibodies, function as antigen-presenting cells that activate pathogenic T cells, and release proinflammatory cytokines that amplify inflammation (1). The clinical gains achieved with antibody therapies have nevertheless exposed fundamental limitations in this treatment approach.

Although anti-CD20 monoclonal antibodies effectively deplete circulating CD20-positive B cells, responses are not uniform across diseases or patients, repeated administration is generally required, and disease activity may recur after B-cell reconstitution. Moreover, CD20 is absent from most terminally differentiated plasma cells, allowing antibody-secreting populations to persist despite effective depletion of circulating B cells (1, 3). In disorders with compartmentalized CNS inflammation, limited penetration of monoclonal antibodies into the CNS may further restrict their effects on tissue-resident immune populations (2, 4). The relevance of this limitation is disease dependent, however, and does not apply equally to disorders centered in the peripheral nervous system or at the neuromuscular junction. These gaps provide a rationale for therapies that can deplete broader B-lineage or plasma-cell compartments, while also underscoring the need to match the intervention to the dominant pathogenic compartment in each disease.

In this context, chimeric antigen receptor (CAR) T-cell therapy is emerging as a promising cellular immunotherapy extending from hematologic malignancies into neuroimmune diseases, where it is being evaluated as a means of achieving potentially deeper depletion of disease-relevant immune-cell populations. Current strategies primarily target CD19 across broad B-cell compartments or B-cell maturation antigen (BCMA) on plasma cells and plasmablasts; these therapeutic targets should be distinguished from disease-associated tissue autoantigens such as AQP4, MOG, acetylcholine receptor, or N-methyl-D-aspartate receptor (2, 5). CAR-T cells can expand after infusion and may reach anatomical compartments that are incompletely accessed by monoclonal antibodies, as suggested by their detection in cerebrospinal fluid in small studies (6, 7). However, cerebrospinal fluid detection does not by itself establish effective trafficking into CNS parenchyma, elimination of tissue-resident pathogenic cells, or restoration of durable immune tolerance.

Clinical experience varies markedly among neurological indications. MG has been studied most extensively, including early-phase studies of conventional and dual-targeted constructs and a randomized, double-blind, placebo-controlled phase 2b trial of a transient BCMA-directed mRNA CAR-T cell platform (8–10). In AQP4-IgG-positive NMOSD, an open-label phase 1 study has reported preliminary feasibility and clinical activity (11). Experience in MS remains limited to small exploratory cohorts of CD19- or BCMA-directed CAR-T cell therapy (6, 12), whereas findings in SPS include individual reports and an open-label phase 2 study reported in abstract form (13–15). Human treatment reports in autoimmune encephalitis and MOGAD remain sparse and do not permit reliable estimates of efficacy or durability (16). Differences in CAR design, lymphodepletion, dosing, outcome definitions, and follow-up further preclude direct comparison across diseases.

Despite encouraging early clinical observations, translation of CAR-T cell therapy into neuroimmunology presents disease-specific biological and safety challenges. For disorders sustained by compartmentalized CNS inflammation, the BBB and local immune milieu may influence CAR-T cell trafficking, persistence, and function, whereas direct CNS entry may be less relevant to diseases centered in the peripheral nervous system or at the neuromuscular junction (17). Whether preexisting neuroinflammation or BBB disruption increases the incidence or severity of immune effector cell-associated neurotoxicity syndrome (ICANS) in patients with neuroimmune diseases remains uncertain (18, 19). Preexisting neurological manifestations may also complicate the recognition and attribution of treatment-related neurotoxicity. In addition, profound depletion of B cells or plasma cells can cause hypogammaglobulinemia, infection, impaired vaccine responses, and other delayed complications, which are particularly relevant in younger patients with non-malignant diseases and long life expectancies (2, 5, 20–22).

Most clinically studied CAR-T cell platforms in neuroimmune diseases currently target B-lineage cells, although approaches directed at other immune-cell populations or designed to promote immune regulation are under investigation. Biomarkers for response and toxicity, validated patient-selection criteria, optimal treatment timing, and standardized disease-specific endpoints remain insufficiently defined. Manufacturing time, scalability, cost, and the uncertain long-term risk–benefit balance further constrain broader clinical use of these therapies (21). The acceptable risk–benefit threshold is particularly difficult to define in refractory but non-fatal neuroimmune diseases. These considerations currently favor evaluation in carefully selected patients with severe, treatment-refractory disease (23).

Against this background, this narrative review examines the biological rationale, clinical applications, and translational challenges of CAR-T cell therapy in neuroimmune diseases. It first outlines CAR design and the immune-cell populations relevant to neurological autoimmunity and then summarizes published clinical experience across MS, NMOSD, MG, SPS, autoimmune encephalitis, MOGAD, and other selected disorders. Comparisons across diseases focus on differences in immunopathogenesis, pathogenic immune-cell compartments, therapeutic targets, CAR platforms, and disease-specific risks. Clinical observations are distinguished from findings derived from preclinical models, biomarker studies, and proposed mechanisms. The review concludes by discussing shared safety concerns, patient selection, practical barriers to implementation, emerging CAR platforms, and priorities for future clinical research.

Relevant publications were identified through targeted searches of PubMed/MEDLINE and ClinicalTrials.gov through July 31, 2026, using combinations of terms related to chimeric antigen receptor T cells, autoimmune or neuroimmune diseases, and the individual neurological disorders covered in this review. Reference lists of relevant primary studies and recent reviews were also screened to identify additional publications. Original reports were preferentially cited when describing patient characteristics, treatment protocols, clinical outcomes, and adverse events, whereas review articles and preclinical studies were used primarily to provide biological and technical context.

## 2 Mechanistic framework and target selection for CAR-T therapy in neuroimmune diseases

CAR-T cell therapy combines cellular engineering with antigen-directed immune-cell depletion. Unlike its use in hematologic malignancies, where the therapeutic objective is eradication of a malignant clone, its application in neuroimmune diseases is intended to remove or regulate non-malignant immune-cell populations that sustain autoantibody production, antigen presentation, or inflammatory signaling. The appropriate target therefore depends on the pathogenic immune compartment involved in each disease, the extent to which that compartment expresses the selected surface antigen, and the consequences of depleting the corresponding normal immune-cell population (5).

### 2.1 Pathogenic immune compartments and target selection

B-cell differentiation encompasses several functionally distinct compartments, including developing and mature B cells, memory B cells, plasmablasts, and short- and long-lived plasma cells. These populations differ in their contributions to antigen presentation, cytokine production, autoantibody secretion, and persistence of immunological memory. Consequently, CAR targets that appear to address the same broad B-cell lineage may deplete substantially different cellular populations (1, 24, 25).

CD19 is expressed from early B-cell development through most mature B-cell stages and is retained by some plasmablasts, but it is generally downregulated during terminal plasma-cell differentiation. CD19-directed CAR-T cells therefore provide broad B-cell depletion and may eliminate part of the plasmablast compartment, while potentially sparing CD19-negative long-lived plasma cells. CD20 has a narrower distribution extending from late pre-B cells to memory B cells and is usually absent from plasmablasts and plasma cells. BCMA is preferentially expressed on plasmablasts and plasma cells, including populations responsible for sustained antibody production, although expression may be heterogeneous. CD38 and CD138 may also permit targeting of antibody-secreting cells, but their broader expression in hematopoietic or non-hematopoietic tissues raises additional concerns regarding target selectivity and off-target toxicity (1, 24).

Disease-associated autoantigens and therapeutic CAR targets are distinct concepts. AQP4 in NMOSD, MOG in MOGAD, and acetylcholine receptor (AChR), muscle-specific kinase (MuSK), and low-density lipoprotein receptor-related protein 4 (LRP4) in MG are targets of pathogenic or disease-associated antibodies; they are not the targets of currently used CD19- or BCMA-directed CAR-T cells (2, 26). Conventional CAR-T strategies instead recognize surface molecules on B-lineage cells or plasma cells. Chimeric autoantibody receptor T-cell platforms differ mechanistically by displaying an autoantigen domain to selectively recognize B cells bearing an autoreactive B-cell receptor, thereby linking disease autoantigen specificity to depletion of the corresponding autoreactive clone (27, 28).

The therapeutic logic is therefore disease dependent. In antibody-mediated disorders such as AQP4-IgG-positive NMOSD and antibody-positive MG, both circulating B cells and antibody-secreting cells may contribute to disease activity, providing distinct rationales for CD19- and BCMA-directed approaches (26). Autoimmune encephalitis may involve variable contributions from peripheral and intrathecal B-cell or plasma-cell compartments according to the associated antibody (29). In multiple sclerosis (MS), B cells may promote disease through antigen presentation, cytokine production, meningeal or perivascular immune organization, and antibody-dependent mechanisms, whereas a single dominant disease autoantigen has not been established (2, 26). The rationale is less direct in stiff-person syndrome, particularly in GAD65 antibody-positive disease, because GAD65 is intracellular and the pathogenic contribution of the antibodies remains uncertain (30). MOGAD is similarly heterogeneous in clinical course and may be monophasic, limiting the population for whom sustained cellular depletion would be justified (31). Target selection should therefore be guided by the dominant pathogenic immune-cell population rather than by the presence of a disease-associated antibody alone.

### 2.2 CAR structure and direct effector mechanisms

A conventional CAR contains an extracellular antigen-recognition domain, a hinge or spacer, a transmembrane region, and an intracellular signaling module that usually combines CD3ζ with one or more costimulatory domains. Binding to a surface antigen activates the CAR independently of major histocompatibility complex presentation and initiates immune-synapse formation, cytoskeletal polarization, cytotoxic granule release, death-receptor signaling, and cytokine production (32). In neuroimmune diseases, these effector functions are directed against non-malignant immune cells; CAR affinity, antigen density, signaling strength, and cellular persistence must therefore be balanced against depletion of protective humoral immunity (5).

The perforin-granzyme pathway mediates rapid target-cell apoptosis after CAR engagement and immune-synapse formation. Perforin facilitates granzyme entry into the target cell, followed by activation of caspase-dependent and caspase-independent death pathways. CAR affinity, surface-antigen density, synapse stability, and intracellular signaling domains influence the efficiency and duration of this response (32, 33). Although this pathway is well established in the biology of CAR-T cells, its relative contribution to depletion of individual pathogenic B-cell and plasma-cell populations in neuroimmune diseases has not been directly defined.

Fas/FasL signaling provides a complementary route to apoptosis and may contribute when granule-mediated cytotoxicity is incomplete (34). Its importance depends on Fas expression and signaling competence in the target population. Activated B cells may be more susceptible than quiescent cells because B-cell activation can upregulate Fas and modify sensitivity to Fas-mediated apoptosis (35). However, the contribution of death-receptor signaling to the depletion of disease-relevant B-cell and plasma-cell subsets has not been established in patients with neuroimmune disease.

Cytokine production constitutes an important link between therapeutic activity and toxicity. High CAR affinity, abundant antigen, and rapid T-cell expansion can generate excessive inflammatory signaling, contributing to cytokine release syndrome (CRS) and endothelial activation associated with immune effector cell-associated neurotoxicity syndrome (32, 36, 37). The timing and magnitude of cytokine responses are affected by CAR design: CD28-containing constructs generally produce more rapid activation and early cytokine release, whereas 4-1BB-containing constructs often exhibit slower expansion and greater persistence (38, 39). In neuroimmune diseases, prolonged persistence may support sustained depletion of pathogenic immune cells but may also extend B-cell aplasia, hypogammaglobulinemia, and infection risk (40). Persistence should therefore not be regarded as uniformly advantageous in a non-malignant setting.

Collectively, these cytotoxic and inflammatory mechanisms determine the depth and duration of target-cell depletion. Their therapeutic consequences depend not only on the CAR construct but also on which B-cell or plasma-cell compartment is targeted and whether prolonged depletion is required for control of the underlying disease.

### 2.3 Effects beyond direct target-cell depletion

Direct antigen-dependent depletion remains the principal intended action of current CD19- and BCMA-directed CAR-T cell platforms in neuroimmune diseases. CAR-T cell activation can nevertheless produce additional local and systemic effects through death-receptor signaling, inflammatory mediators, interactions with endogenous immune cells, and changes in tissue immune activity. These processes have been characterized primarily in tumor systems and preclinical models, and their contribution to treatment responses in human neuroimmune diseases remains uncertain (2, 5, 41–43).

#### 2.3.1 Bystander effects and local inflammation

Bystander killing refers to the elimination of neighboring antigen-negative cells following CAR-T cell activation against an antigen-positive target. In tumor models, localized Fas/FasL signaling can contribute to the death of antigen-negative cells and improve clearance of antigenically heterogeneous tumors (34). CAR-T cell activation may also expose surrounding cells to cytokines and other inflammatory mediators, although cytokine-associated tissue injury should be distinguished from direct CAR-mediated cytotoxicity (36, 37).

Antigen density and CAR affinity influence the activation threshold and magnitude of CAR-T cell responses (44). However, an optimal affinity range for depletion of non-malignant B-lineage cells has not been defined, and the proposal that moderate-affinity constructs are inherently safer in neuroimmune diseases is not supported by comparative clinical studies. Moreover, direct bystander destruction of neurons, astrocytes, or oligodendrocytes has not been demonstrated in patients receiving CAR-T cell therapy for neuroimmune disease. At present, the principal concern is indirect injury associated with systemic or CNS inflammation rather than established antigen-independent killing of neural cells (2, 5, 36).

#### 2.3.2 Antigen spreading

Antigen spreading occurs when immune responses initiated against one antigen promote endogenous responses against additional antigens. In a vaccine-boosted CAR-T solid-tumor model, interferon-γ released by CAR-T cells promoted dendritic-cell recruitment and tumor-antigen uptake, followed by priming of endogenous T cells against antigens not recognized by the original CAR (41, 45). These findings demonstrate that engineered CAR-T cell activation can induce antigen spreading under defined experimental conditions, but they do not establish it as a routine consequence of CAR-T cell therapy.

Antigen spreading has not been directly demonstrated as a mechanism of remission in patients treated with CAR-T cells for neuroimmune disease. Its proposed ability to broaden the elimination of autoreactive lymphocytes therefore remains hypothetical. Conversely, the possibility that extensive tissue injury could expose additional self-antigens and expand autoreactivity is biologically plausible but has not been documented as a clinical complication of CAR-T therapy in this setting (2, 5). Antigen spreading should consequently be regarded as a potential immunological consequence requiring investigation rather than as an established therapeutic or pathogenic mechanism.

#### 2.3.3 Interactions with the local immune environment

Activated CAR-T cells release cytokines and chemokines that can influence endothelial cells, monocytes, macrophages, and other endogenous immune populations. In oncology, these interactions may enhance antitumor activity but also contribute to CRS and immune effector cell-associated neurotoxicity syndrome (36, 37, 42). The consequences may differ in neuroimmune diseases because the relevant target cells, anatomical compartments, and preexisting inflammatory environments vary among indications.

Preclinical studies implicate CNS-resident myeloid cells in neuroinflammation associated with CAR-T cell therapy. In mouse models of ICANS, microglial activation contributed to neurological toxicity, whereas inhibition of microglial TGFβ-activated kinase 1 attenuated inflammatory changes (42). Separate mouse models of CAR-T cell therapy for CNS and non-CNS malignancies demonstrated persistent white-matter microglial reactivity, disrupted oligodendroglial homeostasis and hippocampal neurogenesis, and cognitive impairment after tumor clearance (43). These observations identify potential mechanisms of delayed CNS toxicity, but the models incorporated malignancy, lymphodepletion, and systemic inflammatory responses and cannot be directly extrapolated to patients with neuroimmune disease.

Human studies have not established that CAR-T cells beneficially remodel microglia, eliminate CNS-resident pathogenic immune populations, disrupt ectopic lymphoid structures, restore regulatory T-cell function, or reconstitute durable immune tolerance. Such effects remain proposed mechanisms that require confirmation through paired blood and cerebrospinal fluid analyses, tissue-based studies where feasible, and longitudinal biomarker assessment (2, 5).

### 2.4 Platform design, persistence, and functional limitations

CAR architecture, the duration of receptor expression, the composition and functional state of the infused T-cell product, and the anatomical distribution of target cells collectively influence the depth and duration of immune-cell depletion. In neuroimmune diseases, these variables must be considered in relation to a different therapeutic objective from that in oncology: pathogenic immune populations should be depleted or regulated without unnecessarily prolonging the loss of protective humoral immunity (5, 32, 46, 47).

#### 2.4.1 CAR architecture and platform choice

First-generation CARs contain CD3ζ signaling without an incorporated costimulatory domain and show limited expansion and persistence. Second-generation constructs add a single costimulatory domain, most commonly CD28 or 4-1BB, and form the basis of most clinically studied products. Third-generation constructs combine two costimulatory domains, whereas newer designs include dual-targeted, logic-gated, inducible, or switch-controlled receptors (32, 47–49). These designs are intended to broaden target coverage or permit greater control over CAR-T cell activity, but their advantages have not been established in neuroimmune diseases.

CD28-containing CARs generally produce more rapid signaling and early effector activity, whereas 4-1BB-containing CARs tend to favor memory differentiation and longer persistence (38, 39). Whether either profile is preferable in neuroimmune disease remains unresolved. Rapid expansion may increase inflammatory toxicity, whereas prolonged persistence may extend depletion of normal B cells or plasma cells. Costimulatory-domain selection should therefore be considered together with the target compartment and the desired duration of depletion.

The method used to introduce the CAR provides another level of control. Stable DNA-based gene transfer, commonly performed with integrating viral vectors, can support sustained receptor expression after a single infusion. In contrast, mRNA engineering produces transient CAR expression and may require repeated administration to maintain target-cell depletion (46). A BCMA-directed mRNA CAR-T cell platform has been evaluated in a randomized, double-blind, placebo-controlled phase 2b trial in generalized MG, demonstrating the clinical feasibility of repeated transient CAR-T cell dosing (10). Transient expression may limit prolonged CAR exposure, but it should not be assumed to confer superior safety independently of the target, dose, dosing schedule, and treatment context.

CAR-based platforms also differ in their intended cellular action. Conventional CD19- or BCMA-directed CAR-T cells broadly deplete the B-lineage or antibody-secreting compartments expressing the selected antigen. Chimeric autoantibody receptor T (CAAR-T) cells display a disease-relevant autoantigen domain and are designed to eliminate B cells bearing the corresponding autoreactive B-cell receptor (27, 28). CAR-engineered regulatory T cells are instead intended to concentrate suppressive activity at selected anatomical or immunological targets and are not primarily designed to mediate cytolysis (50). These representative approaches and their manufacturing principles are summarized in **Figure 1**.

**Figure 1:**
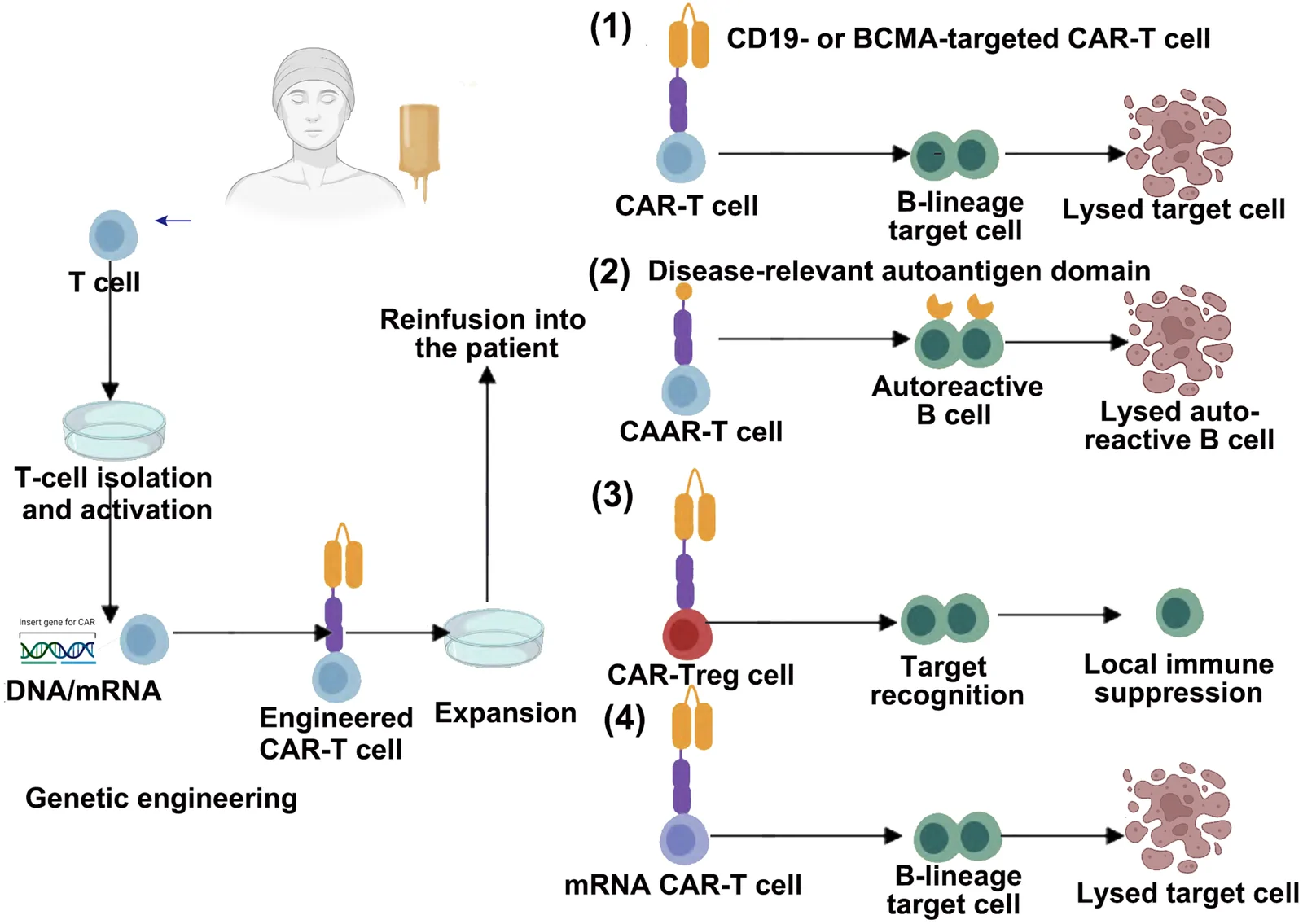
Manufacturing workflow and representative CAR-based platforms for neuroimmune diseases. Autologous T cells are isolated and activated, engineered ex vivo using stable DNA-based or transient mRNA-based methods, expanded as required, and reinfused into the patient. Four representative platforms are illustrated: (1) conventional CD19- or BCMA-directed CAR-T cells, which recognize and lyse antigen-expressing B-lineage target cells; CD19 provides broad B-cell-lineage coverage, whereas BCMA is preferentially expressed by plasmablasts and plasma cells; (2) chimeric autoantibody receptor T (CAAR-T) cells, which display a disease-relevant autoantigen domain to recognize and eliminate B cells bearing the corresponding autoreactive B-cell receptor; (3) CAR-engineered regulatory T (CAR-Treg) cells, which are designed to produce localized immune suppression after target recognition rather than direct target-cell lysis; and (4) mRNA CAR-T cells, which express the CAR transiently and therefore permit time-limited activity and repeated dosing. The platforms differ in target-cell coverage, duration of CAR expression, and intended effector function.

#### 2.4.2 Persistence, differentiation, and functional fitness

The differentiation state of the infused T-cell product influences expansion, immediate effector activity, and persistence. Less differentiated naïve, stem-cell-memory, and central-memory populations generally have greater proliferative potential, whereas effector-memory and terminal effector populations provide more immediate cytotoxic activity but tend to persist for shorter periods. These relationships have been characterized predominantly in oncology and preclinical systems (51). The optimal product composition for neuroimmune diseases is unknown, and prolonged persistence is not necessarily desirable when the target is a normal immune-cell population. Sustained activity may extend B-cell aplasia, hypogammaglobulinemia, and infection susceptibility (40).

CAR signaling, repeated antigen exposure, costimulatory-domain selection, and T-cell differentiation are linked to cellular metabolism and exhaustion. Persistent or antigen-independent signaling can promote mitochondrial dysfunction and expression of inhibitory receptors, thereby reducing CAR-T cell function in tumor and preclinical models (52, 53). Nutrient deprivation and hypoxia can impose additional metabolic constraints in solid tumors (54, 55), but these conditions should not be assumed to characterize the CNS broadly or to determine CAR-T function across neuroimmune diseases.

Metabolic interventions such as enhancement of mitochondrial biogenesis or oxidative phosphorylation remain experimental optimization strategies. No clinical study has demonstrated that metabolic engineering improves CAR-T cell trafficking, persistence, or therapeutic activity in neuroimmune disease (2, 5). Accordingly, the relevance of metabolic engineering to CAR-T therapy for neuroimmune diseases remains uncertain.

### 2.5 Tissue trafficking and the disease-dependent relevance of CNS access

The blood–brain barrier (BBB) restricts, but does not completely prevent, lymphocyte trafficking into the CNS. Immune-cell entry is a regulated, multistep process involving endothelial activation, chemokine signaling, and interactions between leukocyte integrins and endothelial adhesion molecules. The routes and permissiveness of entry differ among vascular, meningeal, and choroid–plexus interfaces and change during neuroinflammation. CAR-T cell distribution therefore cannot be inferred solely from a binary assessment of BBB integrity (56).

The therapeutic relevance of CNS trafficking is disease dependent. In MS and some forms of autoimmune encephalitis, immune activity within the CSF, meninges, perivascular spaces, or brain parenchyma may make access to CNS-associated compartments relevant. In NMOSD and MOGAD, however, depletion of peripheral B-lineage or antibody-secreting cells may reduce pathogenic antibody production and limit replenishment of autoreactive cells without requiring extensive CAR-T cell activity within the brain parenchyma. In MG and Lambert–Eaton myasthenic syndrome, where the principal sites of antibody-mediated injury lie outside the CNS, therapeutic activity would be expected to arise predominantly from depletion of pathogenic immune-cell compartments in the periphery (2, 26).

Human observations should also be interpreted according to anatomical compartment. Detection of CAR-T cells in the CSF after intravenous infusion demonstrates access to the CSF compartment but does not, by itself, establish entry into the brain parenchyma, direct elimination of CNS-resident immune cells, or disruption of meningeal ectopic lymphoid structures. In the initial report of two patients with progressive MS, CAR-T cell expansion in the CSF and changes in intrathecal immunological measures supported CNS-associated activity, but the precise anatomical site and mechanism of action remained unresolved (6).

Greater CNS trafficking is therefore not an unqualified therapeutic objective. In oncology cohorts, endothelial activation and increased BBB permeability have been associated with severe neurotoxicity related to CAR-T cell therapy. Strategies intended to enhance CNS access must consequently be evaluated together with measures of systemic inflammation, endothelial injury, and neurological safety (57).

The mechanistic framework outlined above links the therapeutic rationale for CAR-T cell therapy to the pathogenic immune compartment, the antigen expressed by that compartment, the selected CAR platform, and the anatomical site at which immune-cell depletion is expected to act. The following disease-specific sections distinguish published human treatment observations from ongoing trials and preclinical or conceptual applications. **Figure 2** summarizes disease-associated autoantigens and sites of tissue injury in selected disorders; these features define disease biology but should not be conflated with the cellular targets of current CD19- or BCMA-directed CAR-T approaches.

**Figure 2:**
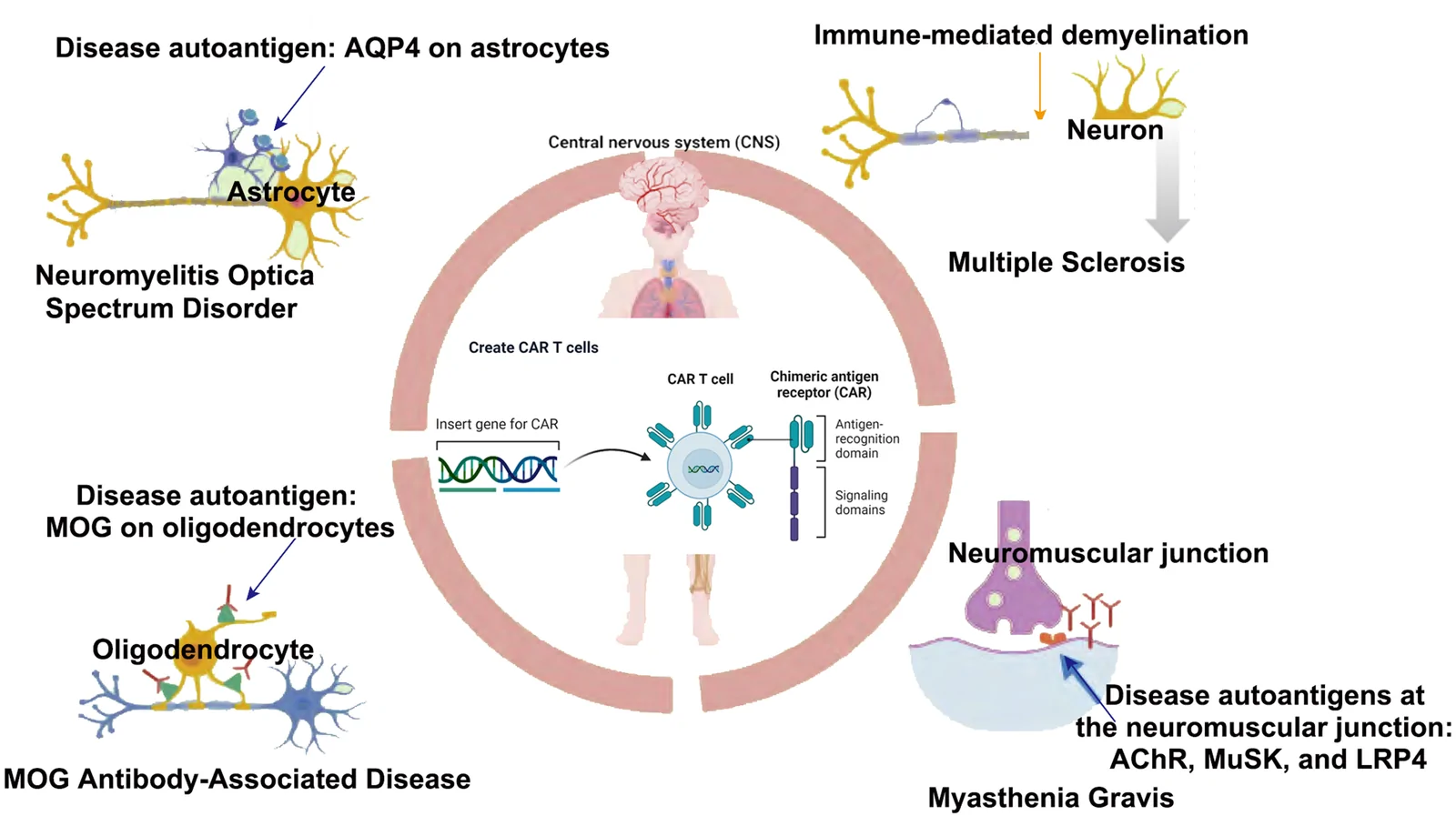
Disease-associated autoantigens and sites of tissue injury in selected neuroimmune diseases. AQP4 on astrocytes in neuromyelitis optica spectrum disorder (NMOSD), MOG on the outer surface of myelin and oligodendrocytes in MOG antibody-associated disease (MOGAD), immune-mediated demyelination in multiple sclerosis (MS), and AChR, MuSK, and LRP4 at the neuromuscular junction in myasthenia gravis (MG) are shown as disease-associated autoantigens or pathological structures. These molecules and tissue structures are not the therapeutic targets of conventional CD19- or BCMA-directed CAR-T cells. Current approaches instead target CD19-expressing B-lineage cells or BCMA-expressing antibody-secreting cells that contribute to pathogenic immunity. The central schematic represents CAR-T cell manufacture and infusion and does not imply direct targeting of astrocytes, oligodendrocytes, neurons, or the neuromuscular junction. AChR, acetylcholine receptor; AQP4, aquaporin-4; BCMA, B-cell maturation antigen; CAR, chimeric antigen receptor; LRP4, low-density lipoprotein receptor-related protein 4; MOG, myelin oligodendrocyte glycoprotein; MuSK, muscle-specific kinase.

## 3 CAR-T cell therapy in multiple sclerosis

Multiple sclerosis (MS) is a chronic immune-mediated disease of the central nervous system (CNS) characterized by inflammation, demyelination, axonal injury, and neurodegeneration. Anti-CD20 monoclonal antibodies substantially reduce relapse activity and new magnetic resonance imaging (MRI) lesions in relapsing MS (58). Ocrelizumab and siponimod slow disability progression in selected populations with primary progressive MS (PPMS) and active secondary progressive MS (SPMS), respectively (59, 60). Progressive disability independent of relapse activity remains a major therapeutic challenge, particularly in non-relapsing progressive MS.

CAR-T cell therapy extends B-cell-directed treatment by enabling sustained depletion of defined B-lineage compartments after a single infusion. Clinical investigation has focused primarily on treatment-refractory progressive MS, in which compartmentalized inflammation, persistent intrathecal immunoglobulin production, and B-lineage populations that are incompletely affected by conventional therapies provide a rationale for targeting CD19 or B-cell maturation antigen (BCMA) (2, 4, 16, 22, 61, 62).

### 3.1 Disease-specific rationale for B-lineage targeting

B cells contribute to MS through antigen presentation, cytokine production, antibody secretion, and organization of inflammatory aggregates in perivascular and meningeal compartments (1, 2, 22, 63). Unlike antibody-mediated disorders defined by a dominant pathogenic autoantibody, MS involves heterogeneous immune responses against multiple CNS antigens. Consequently, CD19- and BCMA-directed CAR-T cell therapies target B-lineage populations rather than a disease-specific autoantigen.

CD20-directed monoclonal antibodies efficiently deplete circulating CD20-positive B cells but spare most antibody-secreting plasma cells and require repeated administration. CD19 is expressed across a broader range of B-cell developmental stages, including plasmablasts and a subset of plasma cells, whereas BCMA is concentrated on plasmablasts and plasma cells. These complementary expression patterns provide distinct therapeutic strategies: CD19-directed CAR-T cells produce broad B-cell depletion, whereas BCMA-directed CAR-T cells preferentially target antibody-secreting compartments (1, 2, 61, 62).

*In vivo* expansion and persistence distinguish CAR-T cells from passive antibody-based depletion. Detection and expansion of CAR-T cells in cerebrospinal fluid (CSF) after treatment further support investigation of these platforms in progressive MS, where immune activity may persist within CNS-associated compartments despite suppression of peripheral inflammatory activity (4, 6, 22).

### 3.2 Published clinical experience with CD19-directed CAR-T cells

In 2024, Fischbach et al. reported the first use of KYV-101, a fully human CD19-directed CAR-T cell product, in two patients with progressive MS who had continued disease progression despite previous therapies. After lymphodepletion with fludarabine and cyclophosphamide, both patients received a single CAR-T cell infusion. Acute tolerability was acceptable, and neither patient developed immune effector cell-associated neurotoxicity syndrome (ICANS). CAR-T cells were detected and expanded in the CSF. In the first patient, reduced intrathecal antibody production was documented and persisted through day 64, linking systemic CD19-directed treatment to measurable changes in the intrathecal immune compartment (6).

Additional clinical experience was reported in a patient with chronic lymphocytic leukemia involving the CNS and concurrent MS. CD19-directed CAR-T cell therapy induced complete remission of the leukemia and was accompanied by a marked reduction in MS disease burden (64). A separate report described relapse of seronegative polyarthritis after CD19-directed CAR-T cell therapy in a patient with concomitant MS, followed by treatment with autologous hematopoietic stem cell transplantation (65). Together, these reports established the clinical feasibility of CD19-directed CAR-T cell administration in patients with MS and documented its capacity to alter systemic and intrathecal B-cell-associated measures.

### 3.3 Early phase 1 experience with BCMA-directed CAR-T cells

BCMA-directed CAR-T cell therapy targets plasmablasts and plasma cells and therefore addresses an immune compartment largely spared by CD20-directed monoclonal antibodies (2, 12, 66, 67). In 2025, Qin et al. reported interim findings from an ongoing phase 1 study of equecabtagene autoleucel, a fully human BCMA-directed CAR-T cell product, in five patients with treatment-refractory progressive MS: one with PPMS and four with SPMS (NCT04561557) (12).

Following lymphodepletion, all five patients received a single CAR-T cell infusion. Serial clinical assessments documented improvement in Expanded Disability Status Scale (EDSS) and performance-based measures, including the Timed 25-Foot Walk and the Nine-Hole Peg Test, during the reported observation period. Plasma cells were depleted in peripheral blood, bone marrow, and CSF-associated compartments, whereas CAR-T cells expanded and persisted in the CSF. CSF κ free light chain concentrations decreased, and oligoclonal bands partially resolved. CSF immune profiling showed attenuation of inflammatory pathways, and 18-kDa translocator protein positron emission tomography (TSPO-PET) demonstrated reduced TSPO signal in the three patients with detectable uptake at baseline (12).

Treatment-associated CRS was limited to grade 1. Grade 3 or higher cytopenias occurred during the first 40 days after infusion, consistent with the combined effects of lymphodepletion and CAR-T cell therapy. These observations linked clinical measures with changes in plasma-cell abundance, intrathecal immunoglobulin production, CAR-T cell kinetics, and CNS-associated inflammatory biomarkers (12).

### 3.4 Mechanistic interpretation: depth, selectivity, and CNS-associated effects

CD19- and BCMA-directed CAR-T cell therapies differ in the B-lineage compartments they deplete, yet both produce a more extensive cellular intervention than repeated administration of a monoclonal antibody. CD19-directed therapy encompasses most mature B cells, memory B cells, plasmablasts, and a subset of plasma cells, whereas BCMA-directed therapy is concentrated on plasmablasts and plasma cells. Neither approach selectively recognizes MS-associated autoreactive clones; their selectivity is determined by lineage-antigen expression (1, 2, 61, 62).

This distinction frames the central therapeutic question in MS: whether clinical activity arises from prolonged peripheral B-lineage depletion, elimination of antibody-secreting cells, access to CNS-associated immune compartments, or the combined effects of lymphodepletion and cellular therapy. Detection of CAR-T cells in the CSF and reductions in intrathecal immunoglobulin measures demonstrate engagement of the CSF-associated immune compartment. The BCMA-directed study further connects these changes with reductions in inflammatory CSF signatures and TSPO-PET signals (6, 12).

The relative depth and cellular specificity of this approach remain active points of discussion. One view emphasizes that sustained depletion across blood, lymphoid tissue, and CNS-associated compartments could control immune activity that persists during conventional treatment. A contrasting view emphasizes the broad removal of normal B-lineage cells and the contribution of lymphodepleting chemotherapy. These positions define the clinical comparison between CAR-T cell therapy, anti-CD20 therapy, and autologous hematopoietic stem cell transplantation (68–70).

### 3.5 Limitations of current clinical observations

The published clinical experience remains limited to two individually treated patients with progressive MS, a patient treated primarily for CNS-involved chronic lymphocytic leukemia, a patient with coexisting rheumatoid arthritis and MS, and five participants in an ongoing phase 1 BCMA-directed study (6, 12, 64, 65). These reports lack concurrent control groups and have heterogeneous clinical contexts, previous treatments, conditioning regimens, outcome assessments, and follow-up durations. They therefore do not establish comparative efficacy against anti-CD20 therapy or autologous hematopoietic stem cell transplantation (16, 68–71).

Changes in EDSS over short observation periods require cautious interpretation because the scale is subject to intraindividual and inter-rater variability, particularly when baseline and follow-up measurements are derived from single assessments (72). Improvement in EDSS or timed performance measures does not identify whether the underlying change reflects suppression of inflammatory activity, neurological recovery, remyelination, or modification of the long-term neurodegenerative process. MRI stability and reductions in inflammatory biomarkers similarly characterize disease activity without directly demonstrating structural neural repair (12).

CSF detection of CAR-T cells, reduced κ free light chains, and partial resolution of oligoclonal bands indicate effects on the intrathecal immune environment but do not demonstrate elimination of B cells or plasma cells from brain parenchyma or meningeal lymphoid aggregates (6, 12, 63). Likewise, the reported TSPO-PET and CSF transcriptional changes do not establish conversion of microglia from an “M1” to an “M2” state, restoration of regulatory T-cell networks, or reestablishment of immune tolerance (12, 73).

The contribution of fludarabine-cyclophosphamide lymphodepletion cannot be separated from that of CAR-T cells in the current uncontrolled reports (12, 16, 62). CD19 and BCMA are B-lineage targets rather than markers restricted to pathogenic clones, and their depletion therefore encompasses normal B cells or plasma cells. The small treated population permits only preliminary characterization of acute safety but cannot quantify uncommon or delayed events. Long-term concerns extrapolated primarily from oncology include sustained hypogammaglobulinemia, opportunistic infection, impaired vaccine responses, prolonged cytopenia, delayed neurotoxicity, secondary malignancy, and uncertain reproductive effects (16, 62, 74–79). These shared safety considerations are examined in the dedicated safety section of this review.

The chronic lymphocytic leukemia case combines the effects of malignancy-directed treatment, lymphodepletion, and leukemia control, whereas the post-CAR-T relapse report concerned seronegative polyarthritis in a patient with concomitant MS who subsequently received autologous hematopoietic stem cell transplantation (64, 65). The predominance of published cases with clinically notable outcomes also makes the current literature susceptible to publication and outcome-reporting bias, particularly because neutral responses, unsuccessful manufacturing, and incomplete follow-up are less likely to appear as individual clinical reports (16).

Several proposed mechanisms remain untested in treated patients with MS. No clinical study has demonstrated disruption of meningeal ectopic lymphoid aggregates, selective elimination of autoreactive clones, restoration of immune tolerance, or preferential clearance of Epstein–Barr virus-infected B cells (6, 12, 63, 73, 80, 81). CAR-T cell persistence in CSF also does not establish direct activity within brain parenchyma. In addition, no biomarker has been validated to predict clinical response to CAR-T cell therapy in MS (71, 82–84).

### 3.6 Ongoing studies and clinical positioning

Registry records available through July 31, 2026, show that prospective development has expanded across several CD19-directed programs. These include a phase 1 study of KYV-101 in non-relapsing progressive MS (NCT06138132), a separate phase 1 study of KYV-101 in treatment-refractory progressive MS (NCT06451159), the phase 2 randomized KYSA-7 study in refractory PPMS and SPMS (NCT06384976), and the phase 1 Breakfree-2 study of CC-97540/BMS-986353 in relapsing and progressive MS (NCT06220201). The BCMA-directed study NCT04561557 continues to provide a complementary strategy centered on antibody-secreting cells (85–87).

These trials incorporate safety, CAR-T cell kinetics, B-lineage depletion, neurological outcomes, MRI measures, and blood or CSF biomarkers (85–87). Baseline characterization can include recent inflammatory activity, intrathecal immunoglobulin synthesis, B-cell and plasma-cell phenotypes, serum and CSF neurofilament light chain, glial fibrillary acidic protein, and imaging measures associated with focal inflammation or progression (12, 71, 82–84). Standardized collection of these measures can clarify the biological contexts associated with treatment response and the timing of CAR-T cell therapy relative to high-efficacy disease-modifying therapies and autologous hematopoietic stem cell transplantation (68–70, 82).

Manufacturing time, previous immunosuppressive exposure, T-cell fitness, product consistency, cost, and access to specialized centers will influence clinical implementation (88). Allogeneic products, transient mRNA CAR-T cells, and *in vivo* CAR generation offer alternative production models, whereas dual-targeted and controllable constructs provide strategies for adjusting depletion depth and CAR-T cell persistence (81, 82). Their relevance to MS will depend on disease-specific clinical development and prospective comparison with established treatment pathways.

CAR-T cell therapy has progressed from individual use of CD19-directed products to prospective evaluation of both CD19- and BCMA-directed strategies in MS. The reported clinical, CSF, cellular, and imaging changes establish a basis for continued clinical development in carefully characterized patients with treatment-refractory progressive disease (6, 12). The ongoing trial portfolio is designed to clarify response durability, the relationship between neurological outcomes and depletion of specific B-lineage compartments, and the clinical position of CAR-T cell therapy within the MS treatment pathway (85–87).

## 4 CAR-T cell therapy in neuromyelitis optica spectrum disorder

NMOSD is a CNS autoimmune disease driven primarily by humoral immunity. In AQP4-IgG-positive NMOSD, autoantibodies bind AQP4 on astrocytic endfeet and initiate complement-dependent inflammation, astrocyte injury, secondary demyelination, and axonal loss. Neurological disability accumulates predominantly through incomplete recovery from attacks involving the optic nerves, spinal cord, brainstem, or other AQP4-rich regions (89). It also differs fundamentally from MOGAD in both pathological mechanisms and clinical course (90). Complement-, CD19-, and interleukin-6 receptor-targeted therapies have substantially improved relapse prevention in AQP4-IgG-positive NMOSD, although recurrent attacks, treatment intolerance, and the need for continued maintenance therapy remain clinically relevant in some patients (91). Anti-CD20 monoclonal antibodies deplete CD20-expressing B cells but largely spare terminally differentiated plasma cells, which can sustain pathogenic antibody production (24). This pathophysiological feature provides a rationale for targeting the antibody-secreting cell compartment with CAR-T cell therapy (2). As illustrated in **Figure 2**, AQP4 is the disease autoantigen expressed on astrocytes, whereas BCMA is a therapeutic CAR target expressed predominantly by plasmablasts and plasma cells; BCMA-targeted CAR-T cells therefore act on pathogenic antibody-secreting cells rather than directly targeting AQP4 or astrocytes (24, 62).

### 4.1 Phase 1 clinical findings of BCMA-targeted CAR-T cell therapy

In 2023, Qin and colleagues reported the interim results of an open-label, single-arm phase 1 clinical trial of the fully human, BCMA-targeted autologous CAR-T cell product CT103A (equecabtagene autoleucel) in patients with relapsed or refractory AQP4-IgG-positive NMOSD (11). A total of 17 patients were screened, 14 underwent apheresis, CT103A was successfully manufactured for 13, and 12 received an infusion. The treated patients had experienced recurrent attacks despite previous maintenance immunotherapy. At data cutoff, the median follow-up was 5.5 months (range, 1–14 months). There were 11 of the 12 patients who remained free from reported relapse after discontinuing corticosteroids and immunosuppressive therapies during the observed follow-up. The median baseline EDSS score was 4.5, and all treated patients had lower EDSS scores at their last reported assessment. Improvements were also reported in visual acuity, ambulation, and bladder function. Serum AQP4-IgG levels decreased in all patients; 7 of 10 evaluable patients were seronegative by week 12, and 5 of 6 were seronegative at month 6 (11). Regarding safety, all patients experienced grade 1–2 CRS, with no grade 3 or higher CRS observed. No ICANS was reported. All 12 patients experienced grade 3 or higher hematologic adverse events during the treatment period, most commonly leukopenia, neutropenia, and lymphocytopenia; CRS resolved without tocilizumab, although three patients received corticosteroids for CRS management (11).

These clinical findings established early human activity of plasma-cell-directed CAR-T cell therapy in relapsed or refractory AQP4-IgG-positive NMOSD and provided the basis for subsequent analyses of CAR-T cell behavior in the CSF (7, 11).

### 4.2 CSF and single-cell observations

To characterize the cellular dynamics associated with CT103A treatment, Qin and colleagues subsequently performed single-cell multi-omic analyses of paired peripheral blood and CSF samples from five consecutive patients treated within the CT103A clinical program (7). CAR-T cell expansion peaked approximately 10 days after infusion and largely declined within the first month. Clonally expanded CAR-T cells were detected in the CSF, and their chemotaxis-associated transcriptional features supported trafficking across the blood-CSF barrier. Proliferating cytotoxic-like CD8^+^ CAR-T cell clones constituted the predominant expanded effector population (7).

Importantly, the study showed that CT103A not only reduced circulating plasmablasts and plasma cells but also depleted these populations in the CSF. These cellular changes were accompanied by reductions in CSF inflammatory signatures and biomarkers of neuroinflammation (7). The analysis also identified a CD44-expressing early-memory phenotype associated with CAR-T cell persistence and a lower expression of cytotoxicity-associated transcriptional programs than that observed in CAR-T cells from patients with hematologic malignancies. Among the five patients included in this analysis, one experienced a relapse with reduced visual acuity on day 45, although serial neurological assessments subsequently improved during longer follow-up (7).

### 4.3 Limitations and NMOSD-specific clinical questions

Interpretation of the CT103A findings is constrained by the open-label, single-arm design, the cohort of 12 treated patients, and the median follow-up of 5.5 months. The patients had highly active disease, with a median interval of 1 month between the most recent attack and infusion, and five patients experienced an attack between screening and treatment. Consequently, changes in EDSS and other functional measures cannot be fully separated from recovery after recent attacks. All patients also received fludarabine- and cyclophosphamide-based lymphodepletion, and the study design does not isolate the contribution of the conditioning regimen to early disease control. In the absence of a comparator, the results do not establish relative efficacy against currently approved relapse-prevention therapies (11, 91).

One major challenge lies in target selection. BCMA provides direct access to plasmablasts and plasma cells but does not cover the broader CD19-expressing B-cell compartment. Conversely, CD19-targeted CAR-T cells cover multiple earlier B-cell stages while variably sparing terminally differentiated plasma cells (16, 24). Comparative clinical activity of these targeting strategies has not been established in NMOSD. Persistent AQP4-IgG after treatment cannot by itself identify BCMA-negative plasma cells, because antibody kinetics may also reflect incomplete target-cell depletion, tissue distribution, CAR-T cell persistence, target density, or assay sensitivity. Dual-target approaches involving B-cell and plasma-cell antigens provide a strategy for broader compartment coverage, although their relevance to NMOSD remains clinically uncharacterized (92).

The duration of observation was insufficient to characterize persistent hypogammaglobulinemia, delayed infections, prolonged cytopenias, or late neurologic events. Preexisting optic nerve, spinal cord, and brainstem injury also complicates the detection and attribution of new neurological symptoms after infusion. These considerations are particularly relevant when distinguishing an NMOSD attack from treatment-related neurotoxicity (66, 93).

AQP4-IgG reduction and seronegativity are informative pharmacodynamic observations but are not validated surrogate endpoints for relapse prevention. Serial AQP4-IgG titers do not consistently predict the occurrence or severity of attacks, and no uniform relapse-associated threshold has been identified (94). Clinical outcomes therefore require separate assessment of adjudicated attacks, disability accumulated through new attacks, and recovery from preexisting neurological deficits. In the available CT103A studies, drug-free, relapse-free status was documented during the observed follow-up, but reversal of established tissue injury or restoration of immune tolerance was not demonstrated (7).

Established relapse-prevention therapies remain the standard clinical approach after a first attack, whereas the risk–benefit profile of CAR-T cell therapy in treatment-naïve or clinically stable patients has not been defined (91).

### 4.4 Clinical positioning and research priorities

Near-term clinical development of CAR-T cell therapy in NMOSD centers on the durability of relapse control, the relationship between immune-cell reconstitution and recurrent disease activity, and its position relative to established targeted therapies. Patients with recurrent AQP4-IgG-positive disease despite adequately administered relapse-prevention therapy, or those unable to maintain such treatment because of serious toxicity, represent the clearest population for prospective investigation (91).

Target development includes broader B-lineage and plasma-cell coverage through alternative or combined targets (92). Within NMOSD, the clinical relevance of these platforms depends on whether residual disease can be linked to a defined pathogenic immune-cell compartment. The presence and persistence of CAR-T cells in blood and CSF, the kinetics of B-cell and plasma-cell reconstitution, and changes in serum and CSF immunoglobulins provide complementary pharmacodynamic measures (7).

Key clinical outcomes include independently adjudicated attacks, time to first relapse, annualized relapse rate, rescue treatment requirements, and sustained changes in visual, motor, sensory, and bladder function. EDSS, disease-specific functional measures, quality of life, AQP4-IgG, immunoglobulin concentrations, and immune-cell reconstitution provide additional clinical and biological context. Standardized outcome ascertainment will permit comparison across CAR targets, conditioning regimens, and existing relapse-prevention therapies (91).

Given the rarity of NMOSD and the small number of patients eligible for intensive cellular therapy, multicenter prospective cohorts and harmonized registries can complement interventional trials by capturing uncommon toxicities, immune reconstitution, late relapse, and long-term functional outcomes (16, 95).

BCMA-targeted CT103A has demonstrated early human feasibility and yielded clinical, serological, and CSF pharmacodynamic observations in relapsed or refractory AQP4-IgG-positive NMOSD (7, 11). Prospective studies using standardized relapse and functional outcomes will define the durability of these findings and the clinical position of CAR-T cell therapy within the expanding NMOSD treatment landscape.

## 5 CAR-T cell therapy in myasthenia gravis

Myasthenia gravis (MG) is an autoantibody-mediated disorder of neuromuscular transmission in which antibodies against the acetylcholine receptor (AChR), muscle-specific kinase (MuSK), low-density lipoprotein receptor-related protein 4 (LRP4), or other components of the neuromuscular junction impair synaptic function. Although complement inhibitors, neonatal Fc receptor inhibitors, and B-cell-directed therapies have expanded the therapeutic options for generalized MG, a subset of patients continues to experience substantial disease activity, treatment-related toxicity, or recurrent need for rescue therapy (96–98). CAR-T cell therapy targets the B-cell populations that generate or sustain these pathogenic autoantibodies rather than the neuromuscular autoantigens themselves. Clinical development in MG now encompasses integrating CD19- and BCMA-targeted products, CD19/BCMA-targeted autologous CAR-T cells, and transient BCMA-targeted mRNA CAR-T cells. These platforms differ in their cellular targets, requirement for lymphodepletion, dosing schedule, duration of CAR expression, and toxicity profile.

### 5.1 CD19-targeted integrating CAR-T cell therapy: case-level experience

CD19 is expressed across most stages of B-cell development and on a proportion of plasmablasts but is generally absent from terminally differentiated long-lived plasma cells. In 2023, Haghikia and colleagues reported the first use of a stably expressed, fully human autologous CD19-targeted CAR-T cell product in a patient with severe, treatment-refractory AChR-antibody-positive generalized MG. Following fludarabine-cyclophosphamide lymphodepletion and a single CAR-T cell infusion, the patient showed marked improvement in walking distance, arm endurance, and MG severity scores by day 62, accompanied by an approximately 70% reduction in the AChR-antibody level (99).

Motte and colleagues subsequently described two women with concomitant MG and Lambert–Eaton myasthenic syndrome who received autologous CD19-targeted CAR-T cell therapy after lymphodepletion. Both regained full mobility within 2 months, in parallel with deep B-cell depletion and normalization of AChR and voltage-gated calcium-channel N-type autoantibodies, and remained clinically stable at 6 and 4 months, respectively (100). A further report described improvement of both AChR-antibody-positive MG and rheumatoid arthritis following CD19-targeted CAR-T cell therapy, accompanied by seroconversion of disease-associated autoantibodies (101).

### 5.2 BCMA-targeted integrating CAR-T cell therapy

BCMA is preferentially expressed by plasmablasts and plasma cells, providing a means of targeting antibody-secreting cells that may escape CD19-directed depletion. Tian and colleagues treated two patients with highly relapsed and refractory MG—one with AChR antibodies and the other with MuSK antibodies—with CT103A, a lentivirally engineered autologous BCMA-targeted CAR-T cell product. Each patient received fludarabine-cyclophosphamide lymphodepletion followed by a single infusion of 1.0 × 10^6^ CAR-T cells/kg. Both achieved minimal symptom expression and sustained clinical improvement for more than 18 months after infusion, whereas pathogenic autoantibody levels declined to very low or nearly undetectable levels and remained suppressed (102).

Longitudinal immune profiling showed depletion of circulating B-cell and plasma-cell populations followed by gradual B-cell repopulation. At 18 months, the reconstituted B-cell compartment was dominated by naïve B cells, whereas switched-memory B cells and plasmablast/plasma-cell populations remained reduced. Single-cell RNA and T-cell receptor sequencing further characterized the expansion and subsequent contraction of cytotoxic CAR-T cell clones and identified functional and metabolic features associated with their relatively limited persistence in these patients (102).

### 5.3 CD19/BCMA-targeted autologous CAR-T cell therapy

Concurrent targeting of CD19 and BCMA broadens B-lineage coverage by engaging both CD19-positive B cells and BCMA-expressing antibody-secreting cells. In 2024, Zhang and colleagues reported a patient with refractory AChR-antibody-positive MG who received bispecific BCMA/CD19-targeted autologous CAR-T cells. Neurological symptoms and circulating AChR antibodies became undetectable after treatment, and the clinical response was maintained without additional immunotherapy during 6 months of follow-up (103).

The subsequent single-arm phase 1 trial enrolled 18 patients with refractory generalized MG who underwent lymphodepleting chemotherapy followed by a single infusion of autologous bispecific BCMA/CD19-targeted CAR-T cells at one of three dose levels. A total of 17 patients completed the day-180 assessment. Mean MG Activities of Daily Living (MG-ADL) and Quantitative Myasthenia Gravis (QMG) scores decreased from baseline by 8.6 and 15.4 points, respectively. Of the 17 patients, 14 achieved minimal manifestation status, 15 discontinued glucocorticoids, all discontinued non-steroidal immunosuppressants, and 8 developed AChR-antibody seronegativity (104).

A longitudinal proteomic and single-cell analysis of six participants from this clinical program showed that five achieved minimal manifestation status without ongoing immunotherapy by month 6 and maintained this status through month 12 despite B-cell repopulation. Reconstituted B cells were predominantly naïve and exhibited reduced AChR specificity and functional activity. In the patient who experienced a disease flare, age-associated B cells expanded and showed increased expression of Fc receptor-like 5 (FCRL5), identifying a relapse-associated B-cell phenotype rather than a population of CD19-negative and BCMA-negative plasma cells expressing CD38 and SLAMF7 (105).

In a separate phase 1 study, Ruan and colleagues administered autologous CD19/BCMA-targeted CAR-T cells without lymphodepletion to six patients with refractory generalized MG. All patients showed clinical improvement, and five achieved an MG-ADL score of 0 by day 90, with responses maintained through days 120-150. All six discontinued glucocorticoids while reducing the use of other immunosuppressants. Longitudinal profiling demonstrated depletion of B cells and plasma cells, transient reductions in memory B cells and plasma cells, attenuation of B-cell receptor signaling in repopulated B cells, and reduced clonal expansion within B- and T-cell compartments (8).

### 5.4 Transient BCMA-targeted mRNA CAR-T cell therapy

Descartes-08 is an autologous BCMA-targeted CAR-T cell product generated by electroporating T cells with CAR-encoding mRNA. The absence of genomic integration results in transient CAR expression, whereas ex vivo T-cell expansion enables administration without lymphodepleting chemotherapy. Unlike the integrating products reported in MG, which were administered as a single infusion, Descartes-08 is delivered as a course of repeated infusions to maintain target engagement during the treatment period (9, 10).

The prospective, multicenter, open-label MG-001 phase 1b/2a study enrolled 14 adults with generalized MG and an MG-ADL score of at least 6. Three participants in the phase 1b component received three ascending doses, whereas 11 participants in the phase 2a component received six doses at the maximum tolerated dose using different dosing schedules. Treatment was administered in the outpatient setting without lymphodepletion. No dose-limiting toxicity, CRS, or neurotoxicity was observed. Among evaluable participants in the phase 2a component, mean changes from baseline at week 12 were −6 points for MG-ADL, −7 for QMG, −14 for the MG Composite (MGC), and −9 for the revised 15-item MG Quality of Life scale (9).

A randomized, double-blind, placebo-controlled phase 2b component of MG-001 subsequently evaluated six once-weekly infusions of Descartes-08. A total of 36 patients were randomized 1:1 to Descartes-08 or placebo and constituted the safety population; the primary efficacy population comprised 26 patients, including 15 assigned to Descartes-08 and 11 to placebo. At month 3, a reduction of at least 5 points in the MGC score—the primary endpoint—was achieved by 66.7% of Descartes-08 recipients and 27.3% of placebo recipients (P = 0.0472). Among AChR-antibody-positive participants, the corresponding proportions were 63.6% and 12.5%. In the Descartes-08 group, 33% achieved minimal symptom expression, defined as an MG-ADL score of 1 or lower, by month 6 and maintained this status through month 12 (10).

Separate long-term follow-up focused on the seven phase 2a participants who received six once-weekly infusions. All seven had clinically meaningful improvement across MG severity measures at month 3, and five maintained the response at month 12. Three participants who subsequently lost clinical benefit received another course of Descartes-08; all improved after retreatment, with two achieving minimal symptom expression that was maintained through 12 months of retreatment follow-up (106).

### 5.5 Immune reconstitution and biomarker findings

Immune profiling across the integrating CAR-T cell studies has documented several patterns of B-cell lineage reconstitution. Following BCMA-targeted CT103A treatment, the B-cell repopulation was dominated by naïve cells, whereas switched-memory B cells and plasmablast/plasma-cell populations remained reduced and pathogenic autoantibodies remained suppressed (102). After BCMA/CD19-targeted CAR-T cell therapy, reconstituted B cells similarly showed naïve predominance, reduced AChR reactivity, and attenuated B-cell receptor signaling (8, 105). In the longitudinal Xuzhou cohort, expansion of FCRL5-expressing age-associated B cells was associated with a disease flare, whereas responders showed persistent reductions in the AChR specificity and functional activity of repopulated B cells (105).

The placebo-controlled biomarker analysis of Descartes-08 identified reductions in BCMA-high plasma cells and activated plasmacytoid dendritic cells, remodeling of the autoantibody repertoire, and changes in selected cytokine and T-cell receptor signatures. These changes occurred without substantial depletion of the broader circulating B-cell compartment or reductions in total immunoglobulin concentrations and vaccine antibody titers (10, 107). Conventional AChR-antibody levels did not differ between treatment groups at month 3 despite the difference in clinical response, indicating that clinical activity and serological change are not invariably concordant (10). In this review, immune reconstitution refers to these documented changes in B-cell and plasma-cell depletion, repopulation phenotype, immune repertoire, and associated soluble biomarkers.

### 5.6 Limitations, MG-specific safety, and clinical positioning

The maturity of the clinical data differs substantially across platforms. Published experience with integrating CD19-targeted CAR-T cell therapy remains confined to individual cases and a two-patient MG/LEMS series (99–101). Integrating BCMA-targeted CT103A has been reported in two patients (102), whereas the BCMA/CD19-targeted programs comprise a case report and small, uncontrolled phase 1 studies (8, 103, 104). MuSK-antibody-positive MG is represented by one patient in the CT103A report, and patients with MuSK antibodies were excluded from the randomized Descartes-08 trial. These datasets therefore do not define comparative activity across MG autoantibody subtypes.

Descartes-08 is the only CAR-T cell platform in MG evaluated in a randomized, placebo-controlled trial. Of 45 eligible patients who underwent leukapheresis, nine did not meet the manufacturing requirement for six therapeutic doses and were treated under a separate open-label protocol; their outcomes were not included in the randomized report. Although 36 patients were randomized, the primary efficacy analysis included 26 participants treated at academic centers who had at least one postinfusion assessment. The primary endpoint was changed from MG-ADL to MGC after enrollment had begun but before unblinding and database lock. Stable background immunosuppressive treatment was permitted, and placebo recipients could cross over to open-label Descartes-08 after the month-3 assessment (10). Consequently, the controlled comparison applies principally to the month-3 endpoint and does not establish superiority over active MG therapies.

Acute toxicity also varies by platform and conditioning regimen. In the lymphodepletion-based BCMA/CD19 phase 1 study, 39% of patients developed grade 1 CRS, no ICANS or dose-limiting toxicity occurred, and the principal grade 3 or higher events were transient hematological abnormalities (104). The two CT103A recipients experienced transient cytopenias; one had asymptomatic cytomegalovirus reactivation, and neither developed ICANS or dose-limiting toxicity (102). In contrast, no CRS or neurotoxicity was reported in the open-label Descartes-08 study, whereas infusion-related reactions were the most frequent adverse events in the randomized trial (9, 10). These platform-specific findings do not support a single pooled estimate of CRS incidence in MG.

MG introduces a distinct clinical safety concern because preexisting bulbar and respiratory muscle weakness can reduce physiological reserve during fever, hypotension, hypoxemia, or other systemic reactions. Risk-adapted management includes assessment of bulbar function and respiratory status before treatment, serial respiratory-function monitoring during the period of expected toxicity, and ready access to ventilatory and intensive-care support for patients with severe baseline weakness or evolving respiratory deterioration (96, 97, 99). Class-wide concerns involving infection, hypogammaglobulinemia, prolonged cytopenias, secondary malignancies, reproductive safety, and long-term surveillance are considered in the subsequent section on shared translational challenges.

The current clinical position of CAR-T cell therapy in MG remains investigational. The populations most closely represented in the integrating CAR-T cell studies are patients with severe generalized MG who have persistent disease activity, repeated crises or rescue-treatment requirements, or unacceptable toxicity despite multiple established therapies. Relevant comparative outcomes include sustained minimal symptom expression, crisis and rescue-treatment frequency, glucocorticoid and immunosuppressant exposure, quality of life, serological changes, treatment-related toxicity, and durability after B-cell repopulation. Direct comparisons with B-cell-depleting antibodies, complement inhibitors, neonatal Fc receptor inhibitors, and other established treatments are not yet available. The published studies nevertheless demonstrate clinical feasibility and early activity across integrating single-target, integrating dual-target, and transient mRNA CAR-T cell platforms, providing a foundation for controlled evaluation of their therapeutic role in refractory MG.

## 6 CAR-T cell therapy in other neuroimmune diseases

Beyond MS, NMOSD, and MG, CAR-T cell therapy has been investigated in several less common neuroimmune disorders involving the CNS, peripheral nerves, and neuromuscular junction. Clinical development is most advanced in stiff-person syndrome (SPS), whereas published experience in MOG antibody-associated disease (MOGAD), autoimmune encephalitis (AE), chronic inflammatory demyelinating polyneuropathy (CIDP), Lambert–Eaton myasthenic syndrome (LEMS), and autoimmune cerebellar ataxia remains heterogeneous. This section distinguishes published human treatment experience from clinical trial reports, preclinical antigen-specific platforms, and neurological disorders reported after CAR-T cell therapy.

### 6.1 Stiff-person syndrome

Stiff-person spectrum disorders are characterized by fluctuating muscle stiffness, painful spasms, gait impairment, and heightened sensitivity to external stimuli. High-titer antibodies against glutamic acid decarboxylase 65 (GAD65) are detected in most patients with classic SPS, whereas glycine receptor and amphiphysin antibodies define additional clinical and immunological subgroups (108). Symptomatic GABAergic agents and immunotherapies, including IVIG, glucocorticoids, plasma exchange, and rituximab, are used in clinical practice, but severe disability may persist in treatment-refractory disease (108).

In 2024, Faissner et al. reported the first use of autologous CD19-directed CAR-T cell therapy in a 69-year-old woman with a 9-year history of treatment-refractory, GAD65 antibody-positive SPS (13). Following lymphodepletion and a single infusion of KYV-101, subsequently named mivocabtagene autoleucel (miv-cel), leg stiffness decreased, walking speed increased by more than 100%, and daily walking distance increased from less than 50 m to more than 6 km within 3 months. Benzodiazepine use was reduced by 40%, and serum GAD65 antibody titers declined from 1:3,200 at baseline to 1:320 by day 144. Treatment-related toxicity was limited to low-grade CRS (13).

A subsequent report described a 62-year-old woman with a 14-year history of GAD65 antibody-positive SPS and concomitant AChR antibody-positive MG (14). Before treatment, she could walk only 10–15 m and experienced up to 30 painful muscle-spasm attacks per day. Following lymphodepletion and a single infusion of CD19-directed CAR-T cells, walking distance increased to 1 km and muscle-spasm attacks decreased to two per day by month 9; GAD65 antibody titers declined from 1:320 to 1:32. Grade 2 CRS and moderate leukopenia occurred, without serious infection. Improvement in stiffness and mobility was accompanied by persistent restriction of elbow extension attributed to established ligament shortening (14).

The multicenter, open-label, single-arm phase 2 KYSA-8 study subsequently evaluated mivocabtagene autoleucel in 26 adults with SPS and an inadequate response to prior immunomodulatory treatment. At a median follow-up of 6.5 months, the median timed 25-foot walk improved by 4.8 s, corresponding to a 45.6% improvement from baseline at week 16; the improvement was sustained at week 24 among the 16 participants who had reached that time point. Low-grade CRS occurred in 92.3% of participants, grade 1 ICANS in 11.5%, and treatment-related grade 3 or 4 neutropenia in 15.4%; all CRS and ICANS events resolved without reported sequelae (15).

GAD65 is an intracellular disease-associated autoantigen and is not the therapeutic target of CD19-directed CAR-T cells. The direct pathogenic contribution of GAD65 antibodies remains debated, and antibody titers do not consistently parallel neurological severity (108). CD19-directed CAR-T cell therapy depletes CD19-expressing B cells, plasmablasts, and some short-lived plasma cells, which may reduce autoantibody production, antigen presentation, and downstream B-cell–T-cell interactions; CD19-negative long-lived plasma cells are not directly targeted (13, 26, 95). The coexistence of GAD65-, glycine receptor-, and amphiphysin-associated phenotypes further indicates that SPS encompasses immunologically distinct subgroups (108).

### 6.2 Myelin oligodendrocyte glycoprotein antibody-associated disease

MOGAD is an inflammatory demyelinating disorder associated with serum IgG1 antibodies against conformational epitopes of MOG. Its clinical phenotypes include optic neuritis, transverse myelitis, acute disseminated encephalomyelitis, and brainstem or cortical encephalitis, and its course may be monophasic or relapsing (90). Recurrent attacks can produce cumulative visual, spinal cord, and cognitive disability (90).

The first published human experience involved a 25-year-old man whose MOGAD began at age 18 and comprised two episodes of myelitis followed by six episodes of left optic neuritis over 6 years despite rituximab and several additional immunotherapies. He received autologous CD19-directed ARI-0001 CAR-T cells after fludarabine-cyclophosphamide lymphodepletion. Complete peripheral CD19-positive B-cell depletion was documented by day 7, followed by predominantly naïve B-cell reconstitution at day 141; memory B cells and plasmablasts remained low or absent during the first year (109).

Serum MOG-IgG became undetectable, but an ipsilateral optic neuritis relapse occurred on day 29, when peripheral B cells remained depleted and MOG-IgG was already negative. The attack did not improve after intravenous methylprednisolone and plasma exchange. No subsequent relapse occurred during approximately 1 year without maintenance immunotherapy, and follow-up MRI and optical coherence tomography remained unchanged. No CRS, ICANS, delayed hematopoietic recovery, or worsening hypogammaglobulinemia was reported (109).

MOG is the disease autoantigen, whereas CD19 is the therapeutic CAR target. CD19-directed CAR-T cells therefore act on B-lineage cells rather than on MOG-expressing oligodendrocytes and do not directly deplete CD19-negative long-lived plasma cells. The persistence of clinical activity after MOG-IgG seronegativity in the reported patient also separates serological conversion from immediate control of CNS inflammation. The subsequent relapse-free period was consistent with control of new inflammatory attacks, whereas the unchanged structural assessments were consistent with persistence of established optic nerve injury (109).

### 6.3 Autoimmune encephalitis

Autoimmune encephalitides comprise a heterogeneous group of inflammatory CNS disorders associated with antibodies against neuronal surface, synaptic, intracellular, or previously uncharacterized antigens. Clinical manifestations include psychiatric and cognitive changes, seizures, movement disorders, cerebellar dysfunction, altered consciousness, and autonomic instability. Disease mechanisms and responses to B-cell-directed therapy vary according to the antigen, the contribution of humoral and cellular immunity, and the presence of an associated tumor (25).

The first published human application involved a 36-year-old man who developed severe, treatment-refractory DAGLA antibody-associated encephalitis after herpes simplex virus type 1 encephalitis. His syndrome was dominated by generalized myoclonus, cerebellar tremor, nystagmus, cognitive impairment, and tetraparesis despite glucocorticoids, plasma exchange, and rituximab. Following fludarabine-cyclophosphamide lymphodepletion, he received a single infusion of 1 × 10^8^ fully human CD19-directed CAR-T cells (KYV-101). Clinical Assessment Scale in Autoimmune Encephalitis and International Cooperative Ataxia Rating Scale scores improved, serum and CSF anti-DAGLA antibodies became undetectable, and CSF oligoclonal bands disappeared. The clinical improvement was maintained at 1 year; reported toxicities included grade 1 CRS and mild hypogammaglobulinemia (110).

Extended follow-up documented sustained clinical stability, absence of relapse, and continued suppression of anti-DAGLA antibodies. Serial volumetric MRI nevertheless demonstrated progressive cerebellar and hippocampal volume loss despite control of B-cell-associated inflammatory activity. This dissociation indicates that suppression of active inflammation and the absence of clinical relapse do not necessarily arrest established or continuing neurodegenerative processes (111).

Antigen-specific cellular therapy has also been examined preclinically in NMDAR encephalitis. NMDAR-specific chimeric autoantibody receptor (CAAR) T cells incorporate an extracellular NMDAR autoantigen domain that recognizes B-cell receptors expressed by NMDAR-reactive B cells. These cells selectively eliminated NMDAR-specific B-cell lines and reduced autoantibody concentrations in a passive-transfer mouse model. This CAAR-T platform is antigen-specific and mechanistically distinct from the broad B-cell depletion produced by conventional CD19-directed CAR-T cell therapy; no human treatment results were reported in that study (112).

### 6.4 Other rare neuroimmune diseases

Human treatment reports have extended to immune-mediated peripheral neuropathies, LEMS, and GAD65 antibody-associated cerebellar ataxia. These disorders involve different pathogenic immune compartments and have been treated with CD19-, BCMA-, or dual CD19/BCMA-directed products.

In 2024, BCMA/CD19 bispecific CAR-T cell therapy was reported in a 44-year-old man with treatment-refractory CIDP. Neurological function improved after a transient early exacerbation, and no relapse was reported during 1 year without maintenance immunosuppression; grade 1 CRS and transient hypotension were observed (113). In a subsequent report, two patients with highly relapsed or refractory CIDP received BCMA-directed CAR-T cell therapy and achieved drug-free remission within 6 months. One relapsed at month 12 after severe COVID-19, accompanied by reemergence of pathogenic immune signatures, whereas the other remained in remission beyond 24 months (114). CD19-directed CAR-T cell therapy has also been reported in a motor-predominant CIDP phenotype and an NF155 antibody-positive autoimmune paranodopathy, with improvement in disability, walking ability, and disease-associated biomarkers (115).

CIDP has also been reported as a delayed neurological complication after BCMA-directed CAR-T cell therapy for multiple myeloma. Two patients developed severe sensorimotor axonal-demyelinating neuropathy after ciltacabtagene autoleucel; one died from respiratory failure and treatment-refractory infection, whereas the other improved after treatment directed at T-cell-mediated inflammation. These cases represent treatment-associated neurological toxicity and are distinct from the therapeutic use of CAR-T cells in preexisting autoimmune neuropathy (116).

An isolated case of severe idiopathic LEMS involved a 54-year-old man with high-titer P/Q-type voltage-gated calcium channel antibodies who received autologous CD19-directed CAR-T cell therapy after lymphodepletion. Treatment produced peripheral B-cell depletion, a reduction in VGCC antibody titers, and an eightfold increase in walking distance. Reported adverse events were grade 2 CRS and intermittent neutropenia (117). Additional cases of overlapping MG and VGCC antibody-positive LEMS are discussed in Section 5.

CD19-directed CAR-T cell therapy was also reported in a 33-year-old man with progressive GAD65 antibody-associated cerebellar ataxia despite rituximab and cyclophosphamide. GAD65 antibody titers decreased by 95% by day 90, clinical improvement was observed by day 30, and no clinical, radiological, or laboratory progression was recorded through day 270. Toxicity was limited to grade 1 CRS (118).

Neuralgic amyotrophy has been reported after CAR-T cell therapy for acute lymphoblastic leukemia (119). This report describes a neurological adverse event after CAR-T cell therapy; no published therapeutic application of CAR-T cell therapy for neuralgic amyotrophy was identified.

### 6.5 Limitations and clinical positioning

The clinical literature summarized in this section remains methodologically heterogeneous. Apart from the open-label, single-arm phase 2 SPS study, published human data consist predominantly of individual cases or small case series with differing disease definitions, CAR targets, lymphodepletion regimens, outcome measures, and follow-up durations (71, 93). The contribution of lymphodepletion cannot be separated fully from that of the infused CAR-T cells in uncontrolled reports, and patient selection and preferential publication of favorable outcomes may inflate apparent treatment effects. No direct comparisons with established biologics, maintenance immunotherapies, or hematopoietic stem-cell transplantation are available. The KYSA-8 results were reported in a conference abstract, and longer-term peer-reviewed follow-up remains unavailable (15).

Clinical outcomes and biomarkers also require separate interpretation. Autoantibody seronegativity does not invariably coincide with immediate suppression of disease activity, as illustrated by the early relapse after CAR-T cell infusion in MOGAD, and control of inflammation does not establish reversal of fixed tissue injury or ongoing neurodegeneration. In AE, the manufacturing interval may be poorly aligned with rapidly progressive disease, whereas preexisting encephalopathy, seizures, movement disorders, and autonomic dysfunction can complicate attribution of post-infusion neurological deterioration to ICANS (25, 120). Across these indications, current observations establish clinical feasibility and early therapeutic activity in selected treatment-refractory patients but do not yet define comparative efficacy, optimal timing, or a routine position within disease-specific treatment algorithms.

The principal published human studies across neuroimmune indications are summarized in **Table 1**, with emphasis on study design, cohort size, follow-up duration, reported clinical outcomes, adverse events, and major interpretive limitations.

**Table 1:** Summary of published human experience with CAR-T cell therapy in neuroimmune diseases.

Disease and CAR platform	Study type and sample size	Follow-up	Main reported outcomes	Main adverse events	Principal limitations	Ref.
MS; CD19-targeted CAR-T cells	Case reports, n=4, in heterogeneous clinical contexts	Intrathecal findings through day 64 in one patient; otherwise NR	CSF CAR-T cell expansion and reduced intrathecal antibody production; improvement in MS-associated disease burden was reported in one patient treated primarily for leukemia	No ICANS in the two patients with progressive MS	Heterogeneous clinical contexts; uncontrolled observations; limited follow-up	(6, 64, 65)
MS; BCMA-targeted CAR-T cells	Phase 1 interim study, n=5	NR	Improvements in EDSS and performance measures; reductions in plasma cells, CSF immunoglobulin-associated measures, inflammatory signatures, and TSPO-PET signals	Grade 1 CRS; grade ≥3 cytopenias	Small, uncontrolled interim cohort	(12)
NMOSD; BCMA-targeted CT103A	Open-label, single-arm phase 1 study, n=12	Median 5.5 months	Eleven patients remained relapse-free after discontinuing immunotherapy; EDSS decreased in all patients; AQP4-IgG seronegativity occurred in 7/10 patients at week 12	Grade 1–2 CRS in all patients; no ICANS; grade ≥3 hematological events in all patients	Single-arm study with short and heterogeneous follow-up	(7, 11)
MG; integrating CD19-targeted CAR-T cells	Individual reports, n=4	Day 62, 4–6 months, or NR	Improvements in mobility, endurance, and MG severity measures, accompanied by B-cell depletion and reductions in disease-associated autoantibodies	NR in the main text	Heterogeneous individual reports	(99–101)
MG; integrating BCMA-targeted CT103A	Case series, n=2	>18 months	Both patients achieved minimal symptom expression with sustained suppression of pathogenic autoantibodies	Transient cytopenias; asymptomatic cytomegalovirus reactivation in one patient; no ICANS	Two-patient uncontrolled experience	(102)
MG; integrating CD19/BCMA-targeted CAR-T cells	One case report; single-arm phase 1 study, n=18; separate phase 1 study without lymphodepletion, n=6	Approximately 4–12 months	Marked reductions in MG-ADL and QMG scores; most evaluable patients achieved minimal manifestation status and reduced or discontinued immunotherapy	Grade 1 CRS in 39% of the lymphodepletion-based phase 1 cohort; no ICANS or dose-limiting toxicity	Small uncontrolled studies using different conditioning and treatment protocols	(8, 103–105)
MG; transient BCMA-targeted mRNA CAR-T cells	Open-label phase 1b/2a study, n=14; randomized phase 2b study, n=36 for safety and n=26 for primary efficacy	12 weeks to 12 months	At month 3, ≥5-point MGC improvement occurred in 66.7% of treated patients and 27.3% of placebo recipients; retreatment restored benefit in three patients who lost response	No CRS or neurotoxicity in the open-label study; infusion-related reactions were most frequent in the randomized study	Small primary efficacy population; manufacturing exclusions; endpoint modification; background immunotherapy and placebo crossover	(9, 10, 106, 107)
SPS; CD19-targeted CAR-T cells	Two case reports; open-label phase 2 study, n=26	Approximately 3–9 months in the case reports; median 6.5 months in the phase 2 study	Improvements in stiffness, walking performance, and spasm frequency; phase 2 study showed a 45.6% median improvement in the Timed 25-Foot Walk at week 16	Low-grade CRS in the first case report; grade 2 CRS and moderate leukopenia in the second case report; in the phase 2 study, low-grade CRS occurred in 92.3%, grade 1 ICANS in 11.5%, and grade 3–4 neutropenia in 15.4%	Phase 2 results available as a conference abstract; no control group	(13–15)
MOGAD; CD19-targeted ARI-0001	Case report, n=1	Approximately 1 year	MOG-IgG became undetectable; optic neuritis relapsed on day 29, followed by approximately 1 year without another relapse	No CRS, ICANS, delayed hematopoietic recovery, or worsening hypogammaglobulinemia	Single case; seronegativity did not coincide with immediate disease control	(109)
DAGLA antibody-associated encephalitis; CD19-targeted KYV-101	Case report, n=1	≥1 year	Clinical improvement, disappearance of serum and CSF anti-DAGLA antibodies and CSF oligoclonal bands, and sustained absence of relapse	Grade 1 CRS and mild hypogammaglobulinemia	Single case; progressive cerebellar and hippocampal volume loss despite inflammatory control	(110, 111)
CIDP; CD19-, BCMA-, or CD19/BCMA-targeted CAR-T cells	Individual reports and small case series	Approximately 1 year to >2 years in reports providing follow-up; NR for the CD19-targeted reports	Improvements in neurological disability and walking ability; drug-free remission was reported in selected patients	Grade 1 CRS and transient hypotension in one report; other safety data incompletely reported	Heterogeneous targets and disease phenotypes; isolated reports and small series	(113–115)
LEMS; CD19-targeted CAR-T cells	Case report, n=1	NR	Reduction in VGCC-antibody titers and an eightfold increase in walking distance	Grade 2 CRS and intermittent neutropenia	Single case	(117)
GAD65 antibody-associated cerebellar ataxia; CD19-targeted CAR-T cells	Case report, n=1	270 days	GAD65-antibody titers decreased by 95%; clinical improvement occurred without recorded clinical, radiological, or laboratory progression	Grade 1 CRS	Single case	(118)

Tips: AQP4, aquaporin-4; BCMA, B-cell maturation antigen; CAR, chimeric antigen receptor; CIDP, chronic inflammatory demyelinating polyneuropathy; CRS, cytokine release syndrome; CSF, cerebrospinal fluid; DAGLA, diacylglycerol lipase-α; EDSS, Expanded Disability Status Scale; ICANS, immune effector cell-associated neurotoxicity syndrome; LEMS, Lambert–Eaton myasthenic syndrome; MG, myasthenia gravis; MG-ADL, Myasthenia Gravis Activities of Daily Living; MGC, Myasthenia Gravis Composite; MOGAD, myelin oligodendrocyte glycoprotein antibody-associated disease; MS, multiple sclerosis; NMOSD, neuromyelitis optica spectrum disorder; NR, not reported; QMG, Quantitative Myasthenia Gravis; SPS, stiff-person syndrome; TSPO-PET, translocator protein positron-emission tomography; VGCC, voltage-gated calcium channel.

## 7 Cross-cutting challenges and clinical translation

Translation of CAR-T cell therapy from early clinical investigation to routine care in neuroimmune diseases requires alignment among target selection, treatment-related risks, patient characteristics, outcome assessment, and manufacturing. These considerations are shared across indications, although their relative importance varies with the pathogenic immune compartment, disease tempo, reversibility of neurological injury, and availability of established therapies (2, 5, 21, 66).

### 7.1 Target breadth, selectivity, and durability

Target selection determines both the pathogenic immune compartment reached by CAR-T cells and the extent of normal humoral immunity removed. CD19-directed CAR-T cells cover most stages of B-cell differentiation and some plasmablasts but do not directly eliminate most CD19-negative long-lived plasma cells. BCMA-directed CAR-T cells more directly deplete antibody-secreting cells but spare upstream memory B cells that can regenerate autoreactive responses. CD20, CD38, and CD138 provide alternative or complementary coverage, although their expression on non-pathogenic immune or non-hematopoietic cells introduces distinct on-target effects. Dual CD19/BCMA targeting is intended to extend depletion across B-cell and plasma-cell compartments but may increase the depth and potential duration of humoral immune suppression (5, 24, 26, 61, 121).

Antigen escape is an established resistance mechanism in B-cell malignancies, whereas treatment failure in neuroimmune diseases may also arise from incomplete coverage of pathogenic immune compartments, limited tissue access, or reemergence of autoreactive clones after immune reconstitution. The central translational objective is therefore to match target breadth and CAR-T cell persistence to disease immunopathology without imposing unnecessary depletion of protective humoral immunity. Antigen-specific platforms, including chimeric autoantibody receptor T cells, are designed to provide greater selectivity for defined autoantibody-mediated diseases, whereas broader B-cell or plasma-cell depletion remains more applicable when the pathogenic repertoire is heterogeneous or incompletely characterized (5, 46, 61, 122).

### 7.2 Safety and long-term surveillance

Acute safety is shaped by both lymphodepletion and CAR-T cell activation. CRS, immune effector cell-associated neurotoxicity syndrome (ICANS), early cytopenias, and infection require standardized assessment and coordinated management by neurology, hematology, intensive care, and infectious disease teams. Baseline neurological examination is particularly important because preexisting weakness, cognitive dysfunction, seizures, movement disorders, or encephalopathy can complicate the recognition of treatment-emergent neurotoxicity. Surveillance during the early postinfusion period includes neurological assessment, blood counts, inflammatory markers, organ function, and evaluation for infection (36, 37, 120, 123).

Long-term toxicity is closely linked to the target and persistence of the CAR-T cell product. Sustained depletion of normal B cells or plasma cells can result in hypogammaglobulinemia, impaired vaccine responses, recurrent infections, and the need for immunoglobulin replacement. Prolonged or delayed cytopenias may further increase infectious risk. Follow-up therefore encompasses blood counts, lymphocyte subsets, immunoglobulin concentrations, infection history, viral reactivation, antimicrobial prophylaxis, and vaccination or revaccination after sufficient immune recovery (124, 125). Delayed neurological symptoms, cognitive function, functional independence, and health-related quality of life are also relevant longitudinal outcomes. Secondary malignancies, including rare T-cell neoplasms, and potential vector-associated clonal expansion are additional surveillance priorities (126). Because many neuroimmune diseases affect children and young adults, counseling regarding fertility preservation, contraception, pregnancy timing, and reproductive follow-up forms part of pretreatment planning (79).

### 7.3 Patient selection and clinical positioning

Candidate selection integrates disease activity, severity, treatment history, reversibility of tissue injury, and correspondence between the dominant pathogenic immune compartment and the proposed CAR target. Assessment of treatment refractoriness incorporates persistent or recurrent clinically meaningful activity despite adequate exposure to established high-efficacy therapies, intolerance that prevents their continued use, or rapid disability accumulation despite appropriate treatment. Fixed neurological disability in the absence of active inflammation represents a different therapeutic context because depletion of pathogenic immune cells does not directly restore established neuronal, axonal, astrocytic, or neuromuscular structural loss. Assessment of inflammatory activity and irreversible injury is therefore central to estimating the potential for relapse prevention and functional improvement (2, 5, 21, 66).

Clinical positioning also depends on disease tempo and available alternatives. Rapidly disabling or life-threatening disease provides a stronger rationale for prospective evaluation of cellular therapy after failure of established treatments, whereas stable non-fatal disease imposes a higher threshold for accepting lymphodepletion and prolonged immune alteration. The timing of CAR-T cell therapy relative to monoclonal antibodies, corticosteroids, intravenous immunoglobulin, plasma exchange, and autologous hematopoietic stem cell transplantation affects infection risk, T-cell collection, manufacturing feasibility, and interpretation of treatment response. In children, adolescents, and patients of reproductive age, the expected duration of benefit must be considered together with decades of potential delayed risk and the feasibility of fertility preservation (5, 21, 61, 95).

### 7.4 Biomarkers and outcome measures

Biomarkers can serve three complementary functions: identifying candidates, monitoring pharmacodynamic effects, and detecting toxicity. Candidate markers include peripheral and cerebrospinal fluid B-cell and plasma-cell phenotypes, autoantibody specificity and titer, intrathecal immunoglobulin synthesis, immune repertoire features, and disease-specific measures of inflammatory activity. Pharmacodynamic monitoring includes CAR-T cell expansion and persistence, depletion and reconstitution of target-cell populations, immunoglobulin recovery, autoantibody kinetics, and changes in autoreactive B-cell or T-cell clonotypes. Single-cell profiling, immune repertoire sequencing, and spatial analyses can characterize immune reconstitution and distinguish broad lymphocyte recovery from reemergence of disease-associated clones; spatial analyses may add tissue-level context where suitable specimens are available (7, 8, 107).

Neurological outcome assessment requires integration of immunological markers with tissue-injury measures and disease-specific clinical scales. Neurofilament light chain and glial fibrillary acidic protein can provide complementary measures of axonal and astrocytic injury, whereas magnetic resonance imaging, optical coherence tomography, electrophysiology, and positron emission tomography address disease-specific structural or inflammatory changes (127, 128). None of these measures alone establishes clinical benefit; longitudinal interpretation links target-cell depletion and immune reconstitution to relapse activity, disability, cognition, quality of life, and treatment-related toxicity. Standardized sampling schedules and assay methods are essential for comparisons across centers and platforms (129).

### 7.5 Manufacturing, access, and regulatory considerations

Autologous CAR-T cell production requires leukapheresis, T-cell activation, genetic modification, expansion, release testing, cryopreservation, transport, lymphodepletion, and reinfusion. Prior immunosuppressive treatment, lymphopenia, systemic inflammation, and disease-related T-cell dysfunction can influence the starting material, although successful manufacturing has been demonstrated across several autoimmune and neuroimmune populations (130, 131). Product evaluation includes identity, viability, transduction efficiency, potency, sterility, replication-competent vector testing, and consistency across manufacturing batches. Standardized chain-of-identity and chain-of-custody procedures are especially important for individualized autologous products (123).

The interval between cell collection and infusion creates a practical constraint in rapidly progressive disease and may require bridging therapy. Allogeneic and *in vivo*-generated CAR-T cell platforms could shorten production or reduce dependence on individualized ex vivo manufacturing, whereas transient mRNA CAR-T cells primarily modify persistence and dosing. Each approach introduces distinct requirements for immunogenicity, persistence, off-target activity, quality control, and regulatory oversight (9, 132–134). Total treatment cost extends beyond product manufacture to lymphodepletion, hospitalization, toxicity management, immunoglobulin replacement, long-term surveillance, and specialized multidisciplinary care. Clinical translation therefore depends on manufacturing capacity, geographic access to accredited centers, reimbursement, and regulatory frameworks adapted to both integrating and non-integrating cellular platforms.

### 7.6 Limitations of the current clinical literature

The current clinical literature is dominated by case reports, small case series, and early-phase single-arm studies. Randomized controlled data remain rare and are confined to selected platforms and indications. Positive outcomes are more likely to be reported than treatment failure, early relapse, manufacturing failure, failure to proceed to infusion, or neutral biomarker findings. Consequently, published cohorts may overrepresent patients with favorable clinical courses (16, 20, 66, 92, 93, 135, 136).

Interpretation is further complicated by heterogeneity in diagnostic criteria, disease phenotype, prior treatment, CAR construct, target, lymphodepletion regimen, outcome definition, and follow-up duration. Clinical improvement after treatment may coincide with recovery from a preceding relapse, concomitant therapy, rehabilitation, or natural fluctuation, whereas changes in autoantibody titers or peripheral target-cell counts do not necessarily correspond to neurological recovery. Cross-study comparisons cannot provide reliable estimates of relative efficacy under these conditions. Prospective registration, consistent reporting of all enrolled and manufactured cases, standardized disease-specific endpoints, appropriate concurrent comparators, and longitudinal documentation of relapse, immune reconstitution, delayed toxicity, cognition, and quality of life are required for interpretable clinical development.

## 8 Conclusions and future directions

CAR-T cell therapy has shown clinical activity in selected patients with refractory neuroimmune diseases, particularly when B cells or plasma cells constitute major pathogenic compartments. Published human experience now includes integrating CD19- and BCMA-directed products, dual-targeted constructs, and transient mRNA CAR-T cells. These platforms differ in target coverage, lymphodepletion requirements, persistence, dosing, and long-term risk and therefore cannot be interpreted as a single therapeutic approach. Clinical maturity also differs among indications, ranging from isolated case reports to prospective early-phase studies and limited randomized data (66, 71, 92, 93).

Current observations do not establish superiority over existing standard therapies, and deep depletion followed by immune reconstitution does not establish cure. Short-term drug-free remission does not establish long-term durability, whereas reduction or disappearance of circulating autoantibodies does not necessarily indicate reversal of established neurological injury. Similarly, the biological rationale of antigen-specific, regulatory, switchable, allogeneic, or *in vivo*-generated CAR-T platforms does not establish their clinical readiness in neuroimmune diseases. These distinctions are particularly important when irreversible neurological disability, slowly progressive neurodegeneration, or effective established therapies influence the expected risk–benefit balance.

The principal research priorities are multicenter prospective studies with appropriate concurrent comparators, standardized disease-specific endpoints, long-term assessment of relapse and delayed toxicity, and integration of biomarkers that distinguish target engagement, immune reconstitution, tissue injury, and clinical recovery. Transparent reporting must include treatment failure, relapse, manufacturing failure, failure to reach infusion, protocol deviations, and long-term adverse outcomes. CAR-T cell therapy has demonstrated preliminary clinical activity in selected refractory neuroimmune diseases, but it remains investigational for most indications.
